# Goal disturbance and emotional symptoms in patients with cancer

**DOI:** 10.1007/s10865-026-00683-1

**Published:** 2026-07-08

**Authors:** G. Elise Doppenberg-Smit, Myra E. van Linde, Femke Lamers, Annemarie M. J. Braamse, Rutger Koornstra, Evelien J. M. Kuip, Mariette Labots, Rune A. W. van de Wetering, Henk M. W. Verheul, Joost Dekker, Adriaan W. Hoogendoorn, Aartjan T. F. Beekman

**Affiliations:** 1 Department of Psychiatry, Amsterdam University Medical Center, Vrije Universiteit Amsterdam, Amsterdam, The Netherlands; 2 Amsterdam Public Health, Mental Health Program, Amsterdam, The Netherlands; 3 Cancer Centre Amsterdam, Cancer Treatment and Quality of Life, Amsterdam, The Netherlands; 4 Department of Medical Oncology, Amsterdam University Medical Center, Vrije Universiteit Amsterdam, Amsterdam, The Netherlands; 5 Department of Medical Psychology, Amsterdam University Medical Center, University of Amsterdam, Amsterdam, The Netherlands; 6 Department of Medical Oncology, Rijnstate Hospital, Arnhem, The Netherlands; 7 Department of Medical Oncology, Radboud Medical Center, Nijmegen, The Netherlands; 8 Department of Anesthesiology, Radboud Medical Center, Pain and Palliative Care, Nijmegen, The Netherlands; 9 Department of Medical Oncology, Slingeland Ziekenhuis, Doetinchem, The Netherlands; 10 Department of Medical Oncology, Erasmus Medical Center, Rotterdam, The Netherlands

**Keywords:** Goal disturbance, Emotional symptoms, Cancer, Adaptation, Indicator

## Abstract

**Background:**

Cancer is a major stressor leading to a wide range of emotions. Emotions, also negative emotions, are essentially adaptive, in the sense that they help patients cope with cancer. Emotions can also be maladaptive, if they are disproportionately severe or persistent, or interfere with functioning. Our overall goal was to explore whether disturbance of life goals provides a fruitful framework for developing indicators that can distinguish between adaptive and maladaptive emotions.

**Objective:**

To test the hypotheses that (1) goal disturbance and emotional symptoms are reciprocally and positively associated to each other; and (2) the association between goal disturbance and emotional symptoms is stronger in patients with maladaptive emotions as compared to patients with adaptive emotions.

**Methods:**

Patients with (non)metastasized disease who recently started systemic treatment (n = 132) filled out Ecological Momentary Assessments 5 times per day for 14 consecutive days in which emotional symptoms and activities related to life goals were documented. The importance of life goals in six domains was assessed with an interview (work/study, disease-related health, lifestyle health, social activities, religious, spiritual and intellectual activities, and leisure). Patients were grouped as having adaptive or maladaptive emotions based on a reference standard (a psychiatric diagnostic assessment, or the subjective need for professional mental healthcare). Linear mixed models were built in R, with either goal disturbance or negative emotional symptoms as dependent variable.

**Results:**

Goal disturbance for social activities and leisure activities was significantly associated with more emotional symptoms. Goal disturbance for disease-related health activities was significantly associated with less emotional symptoms. No significant association was found for other activities (with one exception). With negative emotions as outcome variable, goal disturbance was more strongly associated with more emotional symptoms in patients with maladaptive emotions than patients with adaptive emotions for social, leisure and lifestyle health activities.

**Conclusion:**

Further research is warranted to develop an indicator of maladaptive emotions in patients with cancer based on the extent of goal disturbance of social, leisure and lifestyle health activities.

**Supplementary Information:**

The online version contains supplementary material available at https://doi.org/10.1007/s10865-026-00683-1.

## Introduction

Patients with cancer often experience a wide range of emotions, such as fear, depression, or anger (National Comprehensive Cancer Network, 2023). Emotions developed over the course of evolution and support humans in their functioning (Frijda, 1986; Tooby & Cosmides, 2008). Emotions, also negative emotions are essentially adaptive: they help humans to adapt to events in their environment, such as the diagnosis and treatment of cancer (Dekker et al., 2017, 2020a, 2020b, 2024). For example, fear causes cognitive, behavioral, and physiological changes which help to face a threatening event. Sadness turns our attention inwards, promoting resignation and acceptance. In some patients, however, emotions are not adaptive. Emotions are maladaptive if they are disproportionally severe or persistent, or if they interfere with functioning (Dekker et al., 2017, 2020a, 2020b, 2024). Examples include severe anxiety that impedes the patient's understanding of the physician's explanation of the treatment, or depression leading to lack of motivation to continue treatment. In this conceptualization, the general pattern of emotional responding is adaptive or maladaptive (rather than a single emotion at a particular moment).

It has been argued that there is a need to develop valid indicators distinguishing between adaptive and maladaptive emotions (Dekker et al., 2017, 2020a, 2020b). Current instruments such as the Distress Thermometer (Tuinman et al., 2008) focus exclusively on the intensity of emotions. Although intensity is indeed an indicator of maladaptive emotions, it is obviously not sufficient: some patients with intense emotions may in fact experience adaptive emotions, which facilitate coping with cancer (Dekker et al., 2017). Patients with adaptive emotions tend to prefer to manage these emotions on their own, with support from family, friends, and clinical staff. Although some may have a need for it, many patients with adaptive emotions decline professional mental health care. On the other hand, referral to professional mental health care is indicated for patients with maladaptive emotions (Dekker et al., 2017, 2020a, 2020b). Because of the differential clinical management, there is a need to develop indicators of maladaptive emotions in patients with cancer, in addition to intensity of emotions. In the present study, we explored whether life goal disturbance provides a fruitful framework for developing such indicators.

Life goals are defined as internal representations of desired states that motivate behavior (Austin & Vancouver, 1996). Life goals give direction to how people live their lives, motivate them to take action, and are important for identity-formation (Hullmann et al., 2016). Strauman hypothesizes that disturbance in the pursuit of life goals results in depressive symptoms (Strauman, 2017, 2021). Strauman built on the well-validated idea of two distinct brain/behavior systems for goal pursuit: the promotion system that operates to maximize positive outcomes, and the prevention system that operates to minimize negative outcomes. Strauman hypothesizes that disturbance in the pursuit of life goals leads to hypo-activation of the promotion system, and thereby to depressive symptoms. Additionally, hypo-activation of the promotion system leads to hyper-activation of the prevention system, resulting in symptoms of anxiety and agitation. Patients with cancer may experience goal disturbance, that is disturbance in the pursuit of life goals due to the disease and its treatment. For example, treatment-induced fatigue may result in loss of the ability to work or to maintain social relationships, and neuropathy can have a major negative effect on efforts to perform daily and leisure activities. Indeed, in patients with cancer life goal disturbance has been found to result in negative emotions such as symptoms of depression and anxiety (Benita et al., 2021; Hullmann et al., 2016; Janse et al., 2016a, 2016b; Offerman et al., 2010). Conversely, symptoms of depression and anxiety may interfere with the pursuit of life goals (Anderson et al., 2023; Leduc-Cummings et al., 2024; Offerman et al., 2010), also in patients with cancer (Haydon et al., 2019; Hullmann et al., 2016). Thus, we hypothesized a bidirectional relationship between life goal disturbance and negative emotions in patients with cancer: life goal disturbance leads to negative emotions, and negative emotions lead to life goal disturbance. To date, this bidirectional relationship has not been examined in a single, longitudinal study.

Strauman (2017, 2021) supposes that the cumulative experience of goal disturbance ultimately results in a tipping point, whereby negative emotions turn into the mental disorder of depression. This theory led us to hypothesize that in patients with cancer and maladaptive emotions the impact of life goal disturbance on negative emotions is stronger, compared to patients with adaptive emotions (Dekker et al., 2024). Conversely, we hypothesized that also the impact of negative emotions on life goal disturbance is stronger in patients with maladaptive emotions, compared to patients with adaptive emotions. In a qualitative study exploring how patients with cancer deal with their emotional symptoms, patients reported to generally accept negative emotions. If negative emotions interfered with reaching a desirable goal (e.g., going back to work), emotions were seen as maladaptive, necessitating professional mental healthcare (Braamse et al., 2017); that is, negative emotions have an impact on goal disturbance, particularly in patients with maladaptive emotions.

The objective of the present study was to test the hypotheses that (1) life goal disturbance and negative emotional symptoms are bidirectionally associated with each other; and (2) that the bidirectional association between goal disturbance and negative emotional symptoms is stronger in patients with maladaptive emotions as compared to patients with adaptive emotions. If these hypotheses would be confirmed, the extent of goal disturbance can be used an indicator of maladaptive emotions in patients with cancer.

## Methods

### Design

The study was an observational, longitudinal study between August 2021 and April 2024 in patients with a solid malignancy at the departments of medical oncology in six Dutch hospitals (two academic and four non-academic). The presence of adaptive versus maladaptive emotions was assessed with a reference standard: (a) psychiatric diagnostic assessment, or (b) the patient’s subjective need for professional mental healthcare. Life goals were rated in an interview. Shortly after these interviews took place (usually the next day), participants filled out Ecological Momentary Assessment (EMA) for two weeks to assess emotional symptoms and activities related to life goals. The study was registered on clinicaltrials.gov (NCT05062447) and was approved by the Medical Ethics Committee of Amsterdam UMC (2020.099-NL72824.029.20). All participants provided written informed consent.

## Sample

Patients were eligible if they received a diagnosis of a malignant solid tumor (metastasized or non-metastasized), started with (neo)adjuvant or first line chemo- or immunotherapy within 3–7 months prior to enrollment, and had a > 3-month life expectancy. Exclusion criteria were: age < 18; no sufficient understanding of the Dutch language; the presence of psychopathology other than mood or anxiety disorder (e.g. lifetime history of bipolar disorder, schizophrenia, major depressive disorder with psychotic features, schizoaffective disorders); severe cognitive impairments (e.g. dementia); (indication of) brain tumor or brain metastases; a history of severe drug or alcohol abuse within the past 6 months; not competent in the use of a mobile phone; and providing no informed consent. Patients were approached by their treating oncologist or nurse (specialist) to participate in this study.

*Screening.* We aimed to include 80 patients with adaptive and 80 with maladaptive emotions. Because the majority of patients experience adaptive emotions, after 80% of patients with adaptive emotions were included, we applied a screening method (see S1 Appendix). No a priori power analysis was conducted.

## Measures

Sociodemographic information was collected using a self-reported online questionnaire developed for this study. Medical information including cancer diagnosis, metastasis (yes/no), timing of diagnosis and first treatment were retrieved from the medical record.

## Emotional symptoms

Momentary emotional symptoms and activities related to life goals were assessed via mobile phone during 14 consecutive days, 5 times per day using EMA. A personalized link to the EMA was sent per text message using the secured server system RoQua (Sytema & van der Krieke, 2013). A fixed assessment protocol was carried out to obtain time-series data with equidistant time points (Janssens et al., 2018). Patients were instructed to respond as quickly as possible to the prompt and were given 60 minutes to respond to give sufficient time (Janssens et al., 2018). A reminder was sent after 30 minutes. The full EMA protocol consisted of 15 (positive and negative) emotional symptoms and 6 cancer-treatment-related symptoms, followed by 5 questions on activities since the last assessment, and where and with whom they were at the moment of the assessment. For current analyses, only the negative emotional symptoms were used (referred to as ‘emotional symptoms’). Emotional symptoms were rated on a 7-point scale (1 = not at all; 7 = very much) and included feeling irritated, upset, listless/apathetic, down, nervous, bored, anxious, and worried. An index of emotional symptoms was created by adding the scores on these items and dividing it by the number of items. The emotions were derived from the Netherlands Study of Depression and Anxiety (NESDA) EMA diary-protocol (Schoevers et al., 2021), and have been widely used in previous studies (Bennik, 2015; Booij, 2015; Mehl & Conner, 2011; Wichers et al., 2012)*.*

## Goal disturbance

Goal disturbance was derived from the importance of life goals and the momentary activities related to life goals.

*Importance of life goals*: The importance of life goals in six domains was rated using the Life Goal interview, (Pinquart et al., 2008, 2009; Thompson & and Pitts, 1993). In this interview, life goals in six domains were assessed: (1) performance, work and study, (2) health goals related to disease and treatment, (3) health goals related to lifestyle, (4) social relationships, (5) religious, spiritual and intellectual activities and (6) leisure and entertainment. The patients rated the importance of each of these six life goal domains (ratings could range from 1 = ‘not at all important’ to 5 = ‘very important’). The interview procedure was as follows: each domain was discussed with the patient, and they were invited to give everyday examples of the life goals in their personal lives during unstructured conversation. These examples aided the patient’s understanding of what each life goal domain could mean for them personally. Subsequently, the patient was asked to rate the importance of the life goal domain. This procedure was repeated until the importance of all six life goal domains were rated. The interviewer specifically mentioned that the importance of a life goal should not depend on whether that goal was achievable at the moment, only on the importance of that particular life goal.

*Activities related to life goals*: Activities related to life goals were assessed using the EMA. At every prompt (70 prompts) patients were asked what activities they had done since the last assessment. The categories for activities were analogous to the life goal domains in the interview: work/study activities (e.g. going to work), disease-related health activities (e.g. going to the hospital for treatment), lifestyle health activities (e.g. going on a walk), social activities (e.g. drinking coffee with friends), religious, spiritual or intellectual activities (e.g. doing meditations) and leisure activities (e.g. reading a book). Two additional options were available: ‘I did not do any activities’ and ‘I could not do any activities even though I wanted to’. Patients could select more than one activity.

**Fig. 1:**
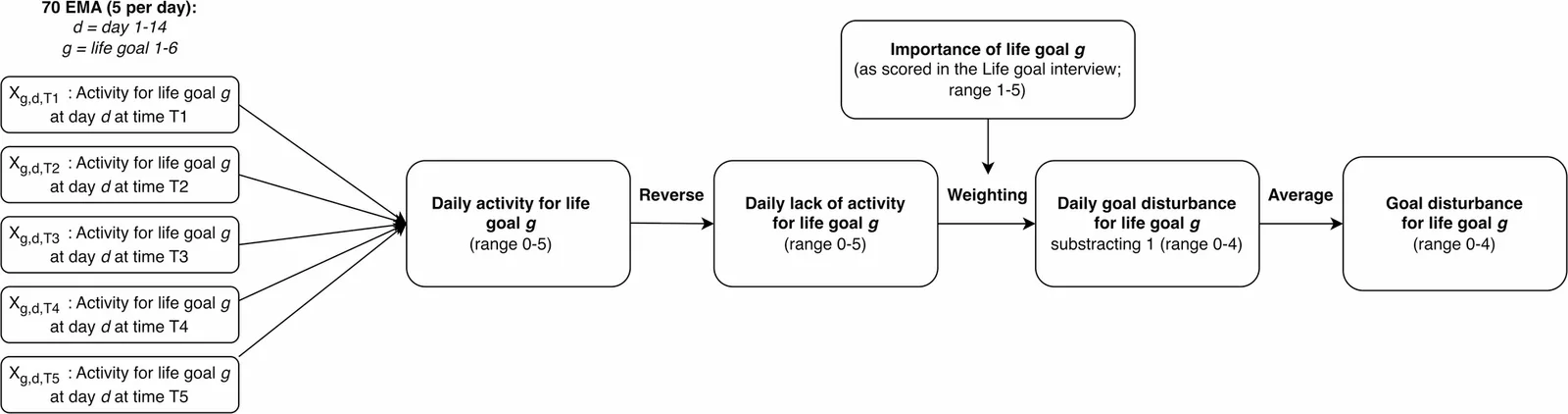
Operationalization of Goal disturbance. Note. EMA = Ecological momentary assessment

*Goal disturbance*: To determine the amount of goal disturbance per life goal, it was first calculated how often the activity related to life goals was done per day (score of maximum 5 per day; 1 for each EMA)(Janse et al., 2016a, 2016b). This resulted in a daily activity score for each of the six life goals. These scores were reversed to indicate a lack of activities related to life goals, rather than the presence. The daily ‘lack of activity score’ was then weighted by the importance of the given life goal as reported in the life goal interview (1 = ‘not at all important’ to 5 = ‘very important’) (Janse et al., 2016a, 2016b; Pinquart et al., 2009). The rationale for this weighting is that unimportant life goals are not disturbed by a lack of activity; only important goals can be disturbed. For interpretive reasons, 1 was subtracted from each goal disturbance score, to make sure a goal disturbance score of 0 would represent no goal disturbance. Goal disturbance had a theoretical range from 0 to 4, where a higher score indicated more goal disturbance. See Fig. 1 for an overview.

## Reference standards

Patients were categorized as experiencing either adaptive or maladaptive emotions, using one of two reference standards. (a) *Psychiatric diagnostic assessment.* The presence of a depressive or anxiety disorder was assessed with the section Anxiety and Depression of the Composite International Diagnostic Interview version 2.1 (CIDI) (Wittchen, 1994). In addition, the presence of an adjustment disorder was assessed with the Distress subscale of the Four Dimensional Symptom Questionnaire (4DSQ) (Kleinstauber et al., 2021), using a cut-off of > 20 on the Distress scale. A one-month recency diagnosis of anxiety or depressive disorder on the CIDI or a score of > 20 on the Distress scale were considered indicators of maladaptive emotions. (b) *Subjective need for professional mental healthcare.* Patients’ subjective need for professional mental healthcare was assessed with the emotional domain of the Problem List (Tuinman et al., 2008). Patients indicated whether in the last three months they experienced a problem in this domain, using a five-point scale (1 = ‘no problem at all’; 5 = ‘very much a problem’) (Braamse et al., 2014; van Linde et al., 2020). When participants reported at least one emotional problem, they were asked whether they had or wished to have a referral to a professional. Maladaptive emotions were rated present if the patient had received a referral or would have preferred to receive a referral for emotional problems (Braamse et al., 2014; van Linde et al., 2020).

## Statistical analyses

Analyses were conducted in R version 4.3.2. (R Core Team, 2021). All variables in the analyses—the scores for emotional symptoms and goal disturbance—were standardized before analyses across the entire sample, to allow the estimates to be interpreted as effect sizes. Descriptive statistics included exploring differences in patients with metastatic and non-metastatic disease. Goal disturbance was analyzed for each life goal domain separately as we expected differences between domains. For each life goal, a linear mixed model with random intercepts for participant ID was built using *goal disturbance as the dependent variable*, with emotional symptoms score and the lagged variables of goal disturbance and emotional symptoms (lag of one day) as independent variables. The lagged variables were included to account for the stability of goal disturbance over time, as well as for any possible delayed effect from emotional symptoms. Another model was built using *emotional symptoms as dependent variable* and the goal disturbance score and the lagged variables as independent variables. Results of both models were summarized in a combined figure. For the comparison of patients with adaptive and maladaptive emotions, the same models were built with the addition of the interaction of group membership (adaptive or maladaptive emotions) with all independent variables. These models were built twice: once for each of the two reference standards. To limit the number of figures, the figures for social activities are presented in the text, and the figures for the other life goals in the supplemental materials. Missings on the EMA were not imputed because emotional symptoms and goal disturbance were calculated as the sum of all measurements of one day (maximum 5), divided by the number of measurements that day. A day was included in the analyses if a patient completed at least one of the five measurements that day.

## Interpretation

For each domain, we tested two hypotheses, each consisting of two sub-hypotheses (stemming from the hypothesized reciprocal/bidirectional association between goal disturbance and emotional symptoms), using one of two reference standards (psychiatric diagnostic assessment and subjective need for professional mental healthcare, which in conceptual terms differ greatly), and testing same day and one day cross-lagged effects. A priori, we decided to interpret the results holistically, focusing on the overall pattern of results rather than on any particular significant effect.

## Results

Of the 134 participants included in the study, 132 patients had sufficient data to be included in the analyses (< 50% missings on the EMA). Of the 1848 theoretically possible observations (14 per patient), 1843 were completed and used in the analyses. Patient characteristics are described in Table 1. Patients with maladaptive emotions had higher emotional symptoms than patients with adaptive emotions according to both reference standards (see Table 2). Goal disturbance differed significantly in five out of twelve models (six life goals times two reference standards), three of which were higher in patients with maladaptive emotions (Table 2). No differences were found in the proportion of males and females in the adaptive and maladaptive group. Regarding age, patients with adaptive emotions according to the subjective need for professional healthcare reference standard were slightly older (see Table S1). Patients with non-metastatic disease experienced somewhat higher emotional symptoms than patients with metastatic disease (1.71 versus 1.61, *t*(1841) = 2.39, *p* = 0.02), while goal disturbance was not consistently higher across the six life goal domains (in three domains higher for patients with metastatic disease, in two domains higher for patients with non-metastatic disease and non-significant difference in one domain).

**Table 1:** Patient characteristics (N = 132)

	n (%) / mean (SD)
Age (range 22–86)	57.7 (12.9)
Gender	
Female	77 (58.3%)
Male	55 (41.7%)
Diagnosis	
Gastro-intestinal cancer	34 (25.8%)
Breast cancer	33 (25.0%)
Melanoma	31 (23.5%)
Urogenital cancer	12 (9.1%)
Gynecological cancer	8 (6.1%)
Sarcoma	7 (5.3%)
Head/Neck cancer	5 (3.8%)
Other	2 (1.5%)
Metastatic disease	
Yes	58 (43.9%)
No	74 (56.1%)
Weeks since diagnosis	62.82 (107.28)
Psychological help in past year	
No	100 (75.8%)
Yes	32 (24.2%)
Reference standard	
Psychiatric diagnostic assessment	
Adaptive emotions	101 (76.5%)
Maladaptive emotions	31 (23.5%)
Subjective need for help	
Adaptive emotions	97 (73.5%)
Maladaptive emotions	35 (26.5%)
Life goals, importance rating (range 1–5)	
Work/study activities	2.83(1.37)
Disease related health activities	4.67(0.61)
Lifestyle health activities	4.36(0.71)
Social activities	4.39(0.87)
Religious/spiritual/intellectual activities	2.99(1.42)
Leisure activities	4.32(0.78)

**Table 2:** Mean and standard deviation of goal disturbance and emotional symptoms by reference standard (N = 132)

	Group	Mean	SD	t-test, p-value
Psychiatric diagnostic assessment				
Goal disturbance				
Work/study activities	Adaptive emotions	1.44	1.26	t(679.94) = -0.33, p = 0.74
	Maladaptive emotions	1.46	1.33	
	Total	1.44	1.27	
Disease-related health activities	Adaptive emotions	3.41	0.85	t(871.02) = -0.28, p = 0.78
	Maladaptive emotions	3.42	0.69	
	Total	3.41	0.82	
Lifestyle health activities	Adaptive emotions	2.96	0.94	t(820.42) = -0.99, p = 0.32
	Maladaptive emotions	3.01	0.80	
	Total	2.97	0.91	
Social activities	Adaptive emotions	2.59	1.08	t(665.91) = 1.83, p = 0.07
	Maladaptive emotions	2.47	1.18	
	Total	2.56	1.10	
Religious, spiritual and intellectual activities	Adaptive emotions	1.69	1.33	t(819.57) = -4.93, p < 0.001
	Maladaptive emotions	2.02	1.14	
	Total	1.77	1.30	
Leisure activities	Adaptive emotions	1.82	1.27	t(807.26) = -1.91, p = 0.06
	Maladaptive emotions	1.94	1.11	
	Total	1.84	1.24	
Emotional symptoms	Adaptive emotions	1.52	0.62	t(560.81) = -14.3, p < 0.001
	Maladaptive emotions	2.18	0.89	
	Total	1.68	0.74	

Subjective need for professional mental healthcare				
Goal disturbance				
Work/study activities	Adaptive emotions	1.57	1.26	t(875.38) = 7.34, p < 0.001
	Maladaptive emotions	1.09	1.24	
	Total	1.44	1.27	
Disease-related health activities	Adaptive emotions	3.40	0.81	t(841.34) = -0.94, p = 0.35
	Maladaptive emotions	3.44	0.83	
	Total	3.41	0.82	
Lifestyle health activities	Adaptive emotions	2.93	0.92	t(901.60) = -3.76, p < 0.001
	Maladaptive emotions	3.10	0.87	
	Total	2.97	0.91	
Social activities	Adaptive emotions	2.62	1.10	t(859.92) = 4.06, p < 0.001
	Maladaptive emotions	2.39	1.10	
	Total	2.56	1.10	
Religious,spiritual and intellectual activities	Adaptive emotions	1.68	1.28	t(839.35) = -5.00, p < 0.001
	Maladaptive emotions	2.02	1.31	
	Total	1.77	1.30	
Leisure activities	Adaptive emotions	1.81	1.24	t(869.36) = -1.83, p = 0.07
	Maladaptive emotions	1.93	1.22	
	Total	1.84	1.24	
Emotional symptoms	Adaptive emotions	1.58	0.67	t(703.44) = -8.14, p < 0.001
	Maladaptive emotions	1.94	0.87	
	Total	1.68	0.74	

For the psychiatric diagnostic assessment, N^adaptive^ = 101 (1412 observations); N^maladaptive^ = 31 (431 observations). For the subjective need for professional mental healthcare, N^adaptive^ = 97 (1356 observations); N^maladaptive^ = 35 (487 observations)

## Association goal disturbance and emotional symptoms

**Fig. 2:**
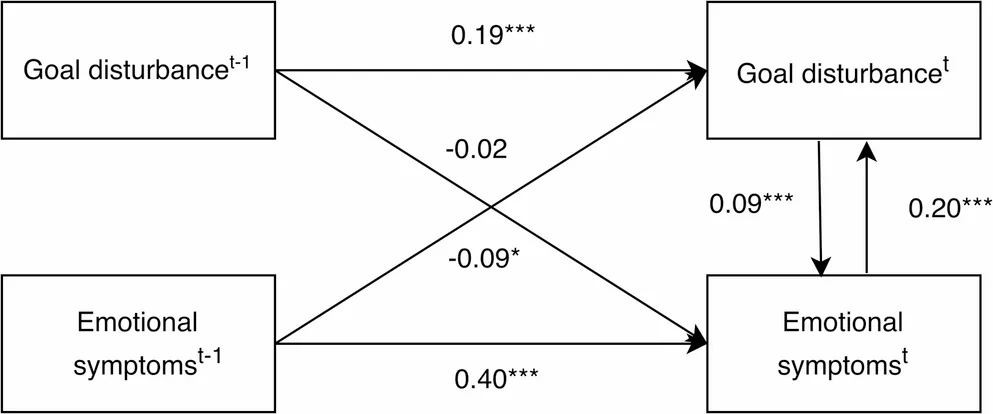
Standardized regression coefficients from two separate linear mixed models on the reciprocal association between goal disturbance of social activities and emotional symptoms. t = day. *Note*: *: *p* < 0.05; **: *p* < 0.01; ***: *p* < 0.001

Analyses from models with emotional symptoms as outcome and those with goals disturbance as outcome showed consistent results. Both models showed that more goal disturbance was significantly associated with more emotional symptoms (conform hypothesis 1) for social activities (Fig. 2) and leisure activities (Fig. S1). Contrary to our hypothesis, the association for disease-related health activities was significant but negative (i.e. more emotional symptoms were associated with less goal disturbance and more goal disturbance was associated with less emotional symptoms; Figure S2). Additionally, the association was not significant for work/study activities (Figure S3), lifestyle health activities (Figure S4) and religious, spiritual and intellectual activities (except for the association with goal disturbance as outcome; Figure S5). With emotional symptoms as outcome variable, only one of the cross-lagged associations from goal disturbance the day before with emotional symptoms was significant (for disease-related health, Figure S2). The range of the associations was − 0.01–0.05; see Figs. 2 and S1–S5). With goal disturbance as outcome variable, more emotional symptoms the day before were associated with more goal disturbance (in the direction of hypothesis 1) for disease-related health activities (Figure S2), but more emotional symptoms the day before were associated with less goal disturbance (not conform hypothesis 1) for social activities and leisure activities (Fig. 2 and S1). For the other three life goals, no significant cross-lagged associations were found (Figure S3–S5). The cross-lagged effects ranged from − 0.13–0.21, which are small effects.

## Adaptive and maladaptive emotions

**Fig. 3:**
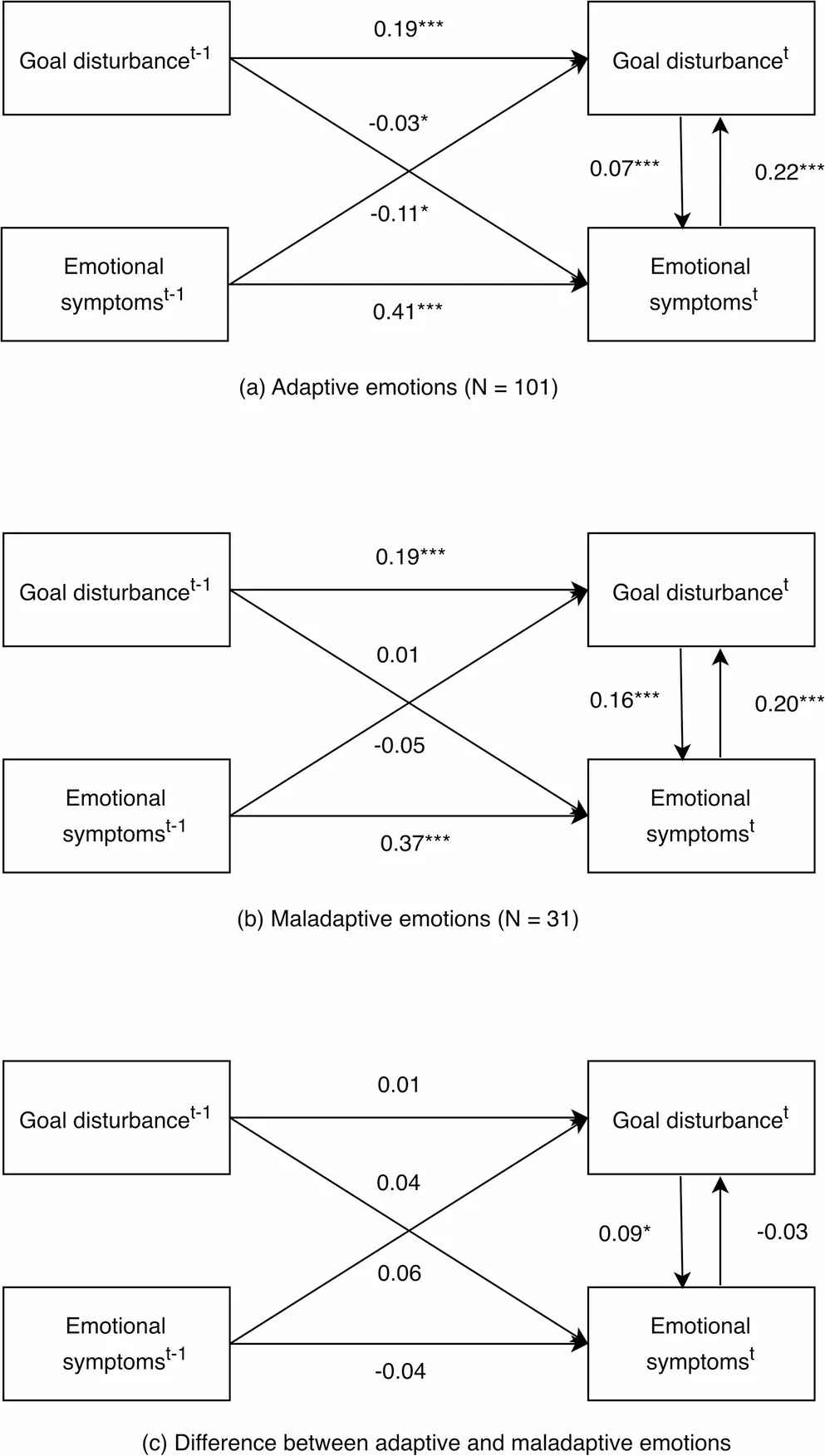
Standardized regression coefficients from two separate linear mixed models on the reciprocal association between goal disturbance of social activities and emotional symptoms with group moderation based on the psychiatric diagnostic assessment reference standard. The top two graphs show parameters for the two separate groups, the bottom graph shows group moderator effects representing differences between the two groups. t = day. *Note*: *: *p* < 0.05; **: *p* < 0.01; ***: *p* < 0.001

**Fig. 4:**
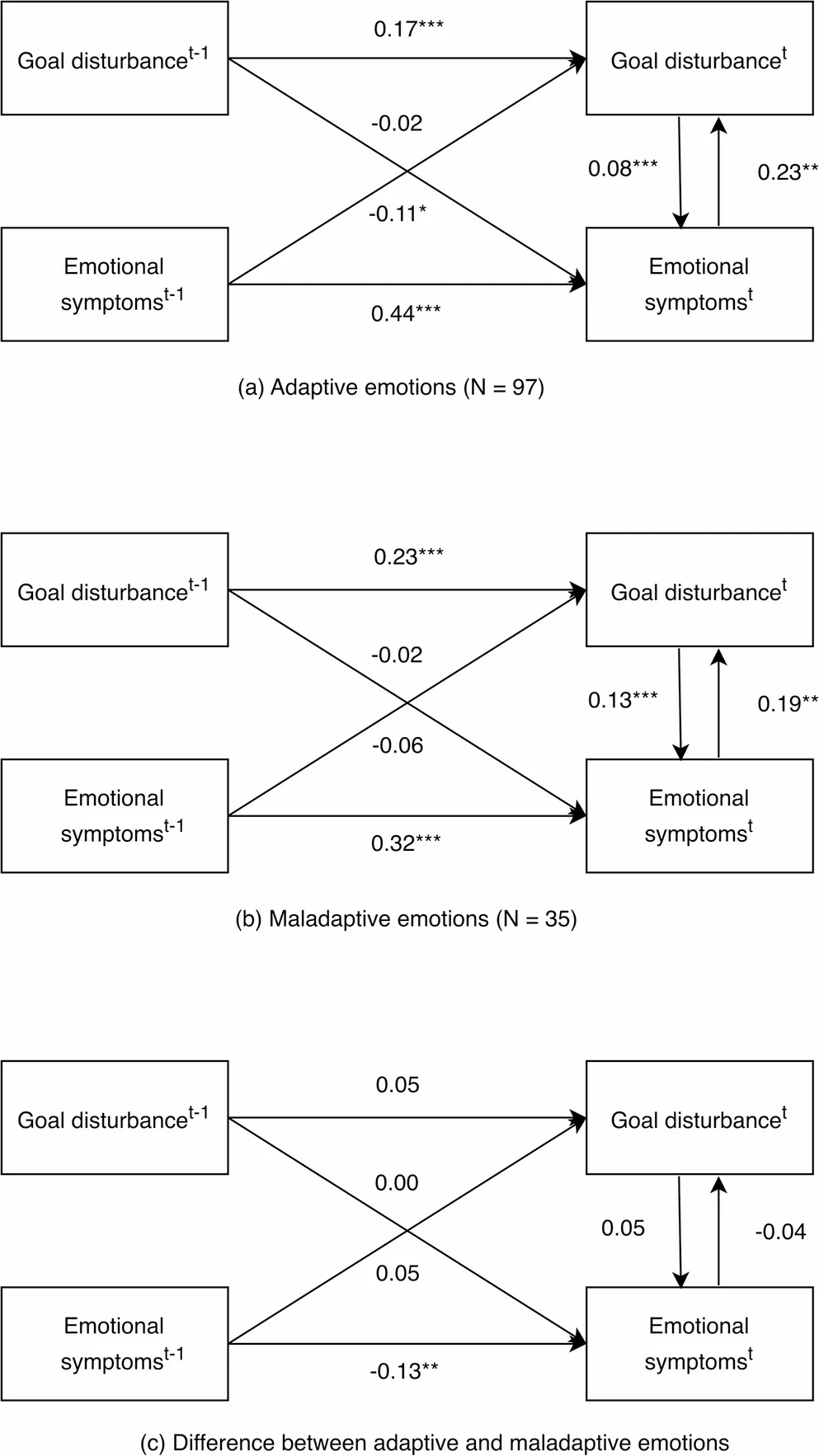
Standardized regression coefficients from two separate linear mixed models on the reciprocal association between goal disturbance of social activities and emotional symptoms with group moderation based on the subjective need for mental healthcare reference standard. The top two graphs show parameters for the two separate groups, the bottom graph shows group moderator effects representing differences between the two groups. t = day. *Note*: *: *p* < 0.05; **: *p* < 0.01; ***: *p* < 0.001

With emotional symptoms as the outcome variable, more goal disturbance was more strongly associated with more emotional symptoms in patients with maladaptive emotional symptoms as compared to patients with adaptive emotions (conform hypothesis 2) in six out of twelve moderation models. These six models included leisure activities (Figure S6 and S7), lifestyle health activities (Figure S8 and S9) and social activities (Fig. 3 and 4), for both reference standards (with one exception: for social activities, the difference between adaptive and maladaptive emotions was in the right direction but non-significant for the subjective need for professional mental healthcare reference standard, see Fig. 4).

Contrary to the hypothesis, more goal disturbance was associated with less emotional symptoms for disease-related health activities, and more so for patients with maladaptive emotions as compared to patients with adaptive emotions (Figure S10 and S11). In the remaining four models (Figure S12–S15), one significant difference was found, for religious, spiritual and intellectual activities according to the psychiatric diagnostic assessment reference standard (Figure S12). In the effect of goal disturbance the day before on emotional symptoms, there were two statistically significant differences across the twelve models. One was stronger for patients with adaptive emotions, and one was stronger for patients with maladaptive emotions.

With goal disturbance as the outcome variable, the effect of emotional symptoms was statistically different in one of the twelve models. That is, the effect was positive and stronger for patients with maladaptive emotions as compared to patients with adaptive emotions (conform hypothesis 2) in the model of lifestyle health using the subjective need for professional mental healthcare as reference standard (Figure S9). No support was found for the hypothesis in the eleven other models. No significant differences were found regarding the effect of emotional symptoms the day before on goal disturbance across the twelve models. The effect sizes of the differences in all 24 moderation models were up to 0.20, which is considered small.

## Discussion

The current study investigated the association between goal disturbance and emotional symptoms in patients with cancer using 14 days of Ecological Momentary Assessment data. The findings confirmed the reciprocal association of goal disturbance and emotional symptoms for social and leisure activities: goal disturbance of social activities and leisure activities was associated with more emotional symptoms, and more emotional symptoms were associated with more goal disturbance of social and leisure activities. In addition, the findings confirmed that more goal disturbance of social, leisure and lifestyle health activities was associated with even more emotional symptoms in patients with maladaptive emotions as compared with adaptive emotions. These findings suggest that further research is warranted to develop an indicator of maladaptive emotions in patients with cancer based on the extent of goal disturbance of social, leisure and lifestyle health activities.

This study showed mixed results: only two out of six life goals showed the positive and reciprocal association between goal disturbance and emotional symptoms. Contrary to our hypothesis 1, the reciprocal association between goal disturbance and emotional symptoms was not found for work/study activities, lifestyle health activities and religious, spiritual and intellectual activities. It is possible that for these life goals the time scale for the impact on each other is longer. Disturbance of life goals related to social activities and leisure activities has a rather immediate effect on emotional symptoms (on the same day) while disturbance of other life goals may have an impact on a longer time scale (e.g., months). A similar argument may hold for the impact of emotional symptoms on life goal disturbance. Further research on this association that uses a longer time scale is recommended. With regard to our second hypothesis and with emotional symptoms as outcome, because no reciprocal association was found, it is not surprising that no support was found for work/study activities, disease-related religious, spiritual and intellectual activities. When using a longer time scale, the hypothesis could still be confirmed. With regard to our second hypothesis and with goal disturbance as outcome, almost no support was found: this hypothesis is clearly rejected.

Surprisingly, for disease-related health activities more goal disturbance was associated with less emotional symptoms (that is, not going to the hospital and not being treated was associated with less emotional symptoms) and vice versa; and more so for patients with maladaptive emotions as compared to patients with adaptive emotions. One possible explanation is that disease-related health is an important life goal, but pursuing it (by going to the hospital and undergoing treatment) does not diminish emotional symptoms, but stirs them up. This finding also raises the question whether our goal disturbance measure is valid for clinician-scheduled activities, as opposed to self-initiated activities.

The cross-lagged effects of emotional symptoms the day before only showed significant associations for three life goals with goal disturbance as the outcome variable. For social and leisure activities, the cross-lagged effect from emotional symptoms the day before it was opposite to the hypothesis; for disease related health-activities it was confirm the hypothesis). Possibly, goal disturbance and emotional symptoms influence each other differently within the same day than between days. It is likely that people make plans based on how they feel. An example may be that when emotional symptoms are high, someone might feel the need to see friends and family the next day for some support (Joo et al., 2020). In addition, it is possible that people with high emotions withdraw from pursuing their goals in other ways than could be captured by current assessment methods. However, because many cross-lagged associations were non-significant and close to zero these may also be coincidental findings.

An overview of the results is provided in Box 1. This overview reveals a clear pattern. Hypothesis 1 on the bidirectional association between goal disturbance and emotional symptoms is confirmed for social and leisure activities, for same-day associations. Hypothesis 2 on the effect of goal disturbance on emotional symptoms is confirmed for social, leisure and lifestyle health activities, for same-day associations, and across both reference standards (with one exception). There were a few other, scattered, confirmatory test results (three for hypothesis 1 and three for hypothesis 2). All other hypothesized associations were not confirmed (either not statistically significant or a statistically significant result contrary to hypothesis). Our a priori decision to interpret the results holistically—focusing on the overall pattern of results rather than on any particular significant effect—led to solid conclusions.

Box 1 *Overview of the results*:

Hypothesis 1

**Table:** 

Same day association		
Activities	Outcome: emotional symptoms*	Outcome: goal disturbance*
Social	✓	✓
Leisure	✓	✓
Lifestyle health	✗	✗
Work/study	✗	✗
Religious, spiritual and intellectual	✗	✓
Disease-related health	⇓	⇓

Cross-lagged association		
Activities	Outcome: emotional symptoms*	Outcome: goal disturbance*
Social	✗	⇓
Leisure	✗	⇓
Lifestyle health	✗	✗
Work/study	✗	✗
Religious, spiritual and intellectual	✗	✗
Disease-related health	✓	✓

* = ✓ hypothesis confirmed =✗ hypothesis not confirmed = ⇓ statistically significant result contrary to hypothesis

Hypothesis 2

**Table:** 

Same day association			
Activities	Reference standard*	Outcome: emotional symptoms**	Outcome: goal disturbance**
Social	1	✓	✗
	2	✗	✗
Leisure	1	✓	✗
	2	✓	✗
Lifestyle health	1	✓	✗
	2	✓	✓
Work/study	1	✗	✗
	2	✗	✗
Religious, spiritual and intellectual	1	✓	✗
	2	✗	✗
Disease-related health	1	⇓	✗
	2	⇓	✗

Cross-lagged association			
Activities	Reference standard*	Outcome: emotional symptoms**	Outcome: goal disturbance**
Social	1	✗	✗
	2	✗	✗
Leisure	1	⇓	✗
	2	✗	✗
Lifestyle health	1	✗	✗
	2	✗	✗
Work/study	1	✗	✗
	2	✗	✗
Religious, spiritual and intellectual	1	✗	✗
	2	✗	✗
Disease-related health	1	✓	✗
	2	✗	✗

*Reference standard 1: Psychiatric diagnostic assessment. Reference standard 2: Subjective need for professional mental health care

** =✓ hypothesis confirmed =✗ hypothesis not confirmed = ⇓ statistically significant result contrary to hypothesis

Our methods to assess goal disturbance and emotional symptoms as well as the reference standards (the psychiatric diagnostic assessment and the subjective need for professional mental healthcare) cover both cancer-related and non-cancer-related issues, without making a distinction. Cancer is a strong stressor, leading to an increased prevalence (Nakash et al., 2014) and incidence (Suppli et al., 2014) of anxiety and depression disorders. In breast cancer, 20–25% of the incidence of depression disorder is attributable to cancer (Dekker et al., 2015). Because cancer is such a strong stressor, including significant goal disturbance, the relationship between goal disturbance and emotional symptoms can be studied well in patients with cancer. It should be noted that both cancer-related and non-cancer-related issues have fed into our results regarding goal disturbance and emotional symptoms. In the field of psycho-oncology, in general, little distinction is made between cancer-related and not-cancer-related issues. For example, the generally recommended assessment of distress (the Distress Thermometer) does not make this distinction. It is quite conceivable that making this distinction will lead to more conclusive research findings. Nevertheless, even without making this distinction, our reference standards and symptom assessments lead to readily interpretable empirical results (Braamse et al., 2014; Doppenberg-Smit et al., 2025; van Linde et al., 2020), possibly because cancer is such a strong stressor. In addition, our goal is to identify indicators of maladaptive emotions during the treatment of cancer. It is well known that pre-existing mental health issues represent a risk factor for distress during cancer treatment (Syrowatka et al., 2017). Clinical experience shows that the distinction between cancer-related and non-cancer-related mental health issues is not clear-cut and that they reinforce each other. From that perspective, if the indicator signals maladaptive emotions, a referral for professional mental health care needs to be considered, irrespective of the exact nature of these emotions.

We used Strauman’s theory on goal disturbance (Strauman, 2017, 2021) to guide the present study, as we considered this theory to be particularly well-suited for distinguishing between adaptive and maladaptive emotions (Dekker et al., 2024). It should be noted though that other theories on goal disturbance (see Carstensen, 2006; Hullmann et al., 2016) and on adaptive and maladaptive emotions (Dekker et al., 2024) exist. Future research could explore the value of these theories in understanding adaptive and maladaptive emotions in patients with cancer.

Strengths of this study include the use of Ecological Momentary Assessment to capture emotional symptoms and activities related to life goals over two weeks. Further, to our knowledge, this study is the first to examine the reciprocal association between goal disturbance and emotional symptoms in patients with cancer in a longitudinal study. Lastly, this study examined life goals across six domains. The findings of this study need to be interpreted in the context of the following limitations. Firstly, the timing of the assessments (3–7 months after start of treatment) may be a good window to distinguish between adaptive and maladaptive emotions as the initial adjustment period after diagnosis is over. However, it is possible that this is still too early, because patients continued to experience the diagnosis and treatment as a major stressor. A second limitation is the uneven sample size between patients with adaptive emotions versus maladaptive emotions. Although patients with maladaptive emotions are a minority, it may be useful to replicate these findings in a more balanced sample. Finally, this study was conducted among Dutch patients who were 3–7 months after start of systemic treatment; it is possible that the results do not generalize to other patient groups.

In conclusion, for social activities and leisure activities, goal disturbance and emotional symptoms were reciprocally associated with each other. In addition, goal disturbance in social activities, leisure and lifestyle health activities was more strongly associated with emotional symptoms in patients with maladaptive emotions as compared to patients with adaptive emotions. Further research is warranted to develop an indicator of maladaptive emotions in patients with cancer based on the extent of goal disturbance of social, leisure and lifestyle health activities.

## Supplementary Information

Below is the link to the electronic supplementary material.

Supplementary file1 (DOCX 34 kb)

Supplementary file2 (DOCX 28 kb)

Supplementary file3 (DOCX 134 kb)

Supplementary file4 (DOCX 780 kb)

## Data Availability

Available upon reasonable request.
