# Supplementary material for: Goal disturbance and emotional symptoms in patients with cancer
Source: J Behav Med. 2026 Jul 8;49(6):1002–13. doi: 10.1007/s10865-026-00683-1 (PMC13748277; doi:10.1007/s10865-026-00683-1)
Supplement: Supplementary file 2 [file 10865_2026_683_MOESM2_ESM.docx]

|  | Group | N | Mean / n | SD / % | Test, p-value |
| --- | --- | --- | --- | --- | --- |
| Psychiatric diagnostic assessment reference standard | | | | | |
| Age | Adaptive emotions | 101 | 58.06 | 12.56 | T(45.44) = 0.488, p = 0.628 |
|  | Maladaptive emotions | 31 | 56.68 | 14.16 |  |
| Females | Adaptive emotions | 101 | 57 | 56.44% | χ^2^ (1) = 0.348, p = -0.556 |
|  | Maladaptive emotions | 31 | 20 | 64.52% |  |
|  | Group | N | Mean / n | SD / % | Test, p-value |
| Subjective need for professional mental healthcare reference standard | | | | | |
| Age | Adaptive emotions | 97 | 60.08 | 12.16 | T(57.39) = 3.542, p < 0.001 |
|  | Maladaptive emotions | 35 | 51.23 | 12.86 |  |
| Females | Adaptive emotions | 97 | 53 | 54.64 | χ^2^(1) = 1.52, p = 0.218 |
|  | Maladaptive emotions | 35 | 24 | 68.57 |  |

S1 Table.

Differences in age and sex between patients with adaptive and maladaptive emotions

N=132
