# Supplementary material for: Goal disturbance and emotional symptoms in patients with cancer
Source: J Behav Med. 2026 Jul 8;49(6):1002–13. doi: 10.1007/s10865-026-00683-1 (PMC13748277; doi:10.1007/s10865-026-00683-1)
Supplement: Supplementary file 3 [file 10865_2026_683_MOESM3_ESM.docx]

Figure S1. Standardized regression coefficients from two separate linear mixed models on the reciprocal association between goal disturbance for leisure activities and of emotional symptoms. t = day. Note. *: *p*<0.05; **: *p*<0.01; ***: *p*<0.001

Figure S2. Standardized regression coefficients from two separate linear mixed models on the reciprocal association between goal disturbance for disease-related health activities and of emotional symptoms. t = day. Note. *: *p*<0.05; **: *p*<0.01; ***: *p*<0.001

Figure S3 Standardized regression coefficients from two separate linear mixed models on the reciprocal association between goal disturbance for work/study activities and of emotional symptoms. t = day. Note. *: *p*<0.05; **: *p*<0.01; ***: *p*<0.001

Figure S4. Standardized regression coefficients from two separate linear mixed models on the reciprocal association between goal disturbance for lifestyle health activities and of emotional symptoms. t = day. Note. *: *p*<0.05; **: *p*<0.01; ***: *p*<0.001

Figure S5. Standardized regression coefficients from two separate linear mixed models on the reciprocal association between goal disturbance for religious, spiritual and intellectual activities and of emotional symptoms. t = day. Note. *: *p*<0.05; **: *p*<0.01; ***: *p*<0.001
