# Supplementary material for: Goal disturbance and emotional symptoms in patients with cancer
Source: J Behav Med. 2026 Jul 8;49(6):1002–13. doi: 10.1007/s10865-026-00683-1 (PMC13748277; doi:10.1007/s10865-026-00683-1)
Supplement: Supplementary file 4 [file 10865_2026_683_MOESM4_ESM.docx]

Figure S6. Standardized regression coefficients from two separate linear mixed models on the reciprocal association between goal disturbance of leisure activities and emotional symptoms with group moderation based on the psychiatric diagnostic assessment reference standard. The top two graphs show parameters for the two separate groups, the bottom graph shows group moderator effects representing differences between the two groups. t = day.

Note. *: *p*<0.05; **: *p*<0.01; ***: *p*<0.001

Figure S7. Standardized regression coefficients from two separate linear mixed models on the reciprocal association between goal disturbance of leisure activities and emotional symptoms with group moderation based on the subjective need for mental healthcare reference standard. The top two graphs show parameters for the two separate groups, the bottom graph shows group moderator effects representing differences between the two groups. t = day. Note. *: *p*<0.05; **: *p*<0.01; ***: *p*<0.001

Figure S8. Standardized regression coefficients from two separate linear mixed models on the reciprocal association between goal disturbance of lifestyle health activities and emotional symptoms with group moderation based on the psychiatric diagnostic assessment reference standard. The top two graphs show parameters for the two separate groups, the bottom graph shows group moderator effects representing differences between the two groups. t = day.

Note. *: *p*<0.05; **: *p*<0.01; ***: *p*<0.001

Figure S9. Standardized regression coefficients from two separate linear mixed models on the reciprocal association between goal disturbance of lifestyle health activities and emotional symptoms with group moderation based on the subjective need for mental healthcare reference standard. The top two graphs show parameters for the two separate groups, the bottom graph shows group moderator effects representing differences between the two groups. t = day.

Note. *: *p*<0.05; **: *p*<0.01; ***: *p*<0.001

Figure S10. Standardized regression coefficients from two separate linear mixed models on the reciprocal association between goal disturbance of disease-related health activities and emotional symptoms with group moderation based on the psychiatric diagnostic assessment reference standard. The top two graphs show parameters for the two separate groups, the bottom graph shows group moderator effects representing differences between the two groups. t = day.

Note. *: *p*<0.05; **: *p*<0.01; ***: *p*<0.001

Figure S11. Standardized regression coefficients from two separate linear mixed models on the reciprocal association between goal disturbance of disease-related health activities and emotional symptoms with group moderation based on the subjective need for mental healthcare reference standard. The top two graphs show parameters for the two separate groups, the bottom graph shows group moderator effects representing differences between the two groups. t = day.

Note. *: *p*<0.05; **: *p*<0.01; ***: *p*<0.001

Figure S12. Standardized regression coefficients from two separate linear mixed models on the reciprocal association between goal disturbance of religious, spiritual and intellectual activities and emotional symptoms with group moderation based on the psychiatric diagnostic assessment reference standard. The top two graphs show parameters for the two separate groups, the bottom graph shows group moderator effects representing differences between the two groups. t = day. Note. *: *p*<0.05; **: *p*<0.01; ***: *p*<0.001

Figure S13. Standardized regression coefficients from two separate linear mixed models on the reciprocal association between goal disturbance of religious, spiritual and intellectual activities and emotional symptoms with group moderation based on the subjective need for mental healthcare reference standard. The top two graphs show parameters for the two separate groups, the bottom graph shows group moderator effects representing differences between the two groups. t = day. Note. *: *p*<0.05; **: *p*<0.01; ***: *p*<0.001

Figure S14. Standardized regression coefficients from two separate linear mixed models on the reciprocal association between goal disturbance of work/study activities and emotional symptoms with group moderation based on the psychiatric diagnostic assessment reference standard. The top two graphs show parameters for the two separate groups, the bottom graph shows group moderator effects representing differences between the two groups. t = day.

Note. *: *p*<0.05; **: *p*<0.01; ***: *p*<0.001

Figure S15. Standardized regression coefficients from two separate linear mixed models on the reciprocal association between goal disturbance of work/study activities and emotional symptoms with group moderation based on the subjective need for mental healthcare reference standard. The top two graphs show parameters for the two separate groups, the bottom graph shows group moderator effects representing differences between the two groups. t = day.

Note. *: *p*<0.05; **: *p*<0.01; ***: *p*<0.001
